# T-Wave Analysis on the 24 h Holter ECG Monitoring as a Predictive Assessment of Major Adverse Cardiovascular Events in Patients with Myocardial Infarction: A Literature Review and Future Perspectives

**DOI:** 10.3390/life13051155

**Published:** 2023-05-10

**Authors:** Ștefania-Teodora Duca, Mihai Roca, Alexandru-Dan Costache, Adriana Chetran, Irina Afrăsânie, Radu-Ștefan Miftode, Ionuț Tudorancea, Iulian Matei, Radu-George Ciorap, Ovidiu Mitu, Minerva Codruța Bădescu, Dan Iliescu-Halitchi, Codruța-Olimpiada Halițchi-Iliescu, Florin Mitu, Cătălina Lionte, Irina-Iuliana Costache

**Affiliations:** 1 Department of Internal Medicine I, Faculty of Medicine, University of Medicine and Pharmacy “Grigore T. Popa”, 700115 Iasi, Romaniadan-alexandru.costache@umfiasi.ro (A.-D.C.);; 2 Department of Cardiology, “St. Spiridon” Emergency County Hospital, 700111 Iasi, Romania; 3 Department of Cardiovascular Rehabilitation, Clinical Rehabilitation Hospital, 700661 Iasi, Romania; 4 Department of Morpho-Functional Science II-Physiology, University of Medicine and Pharmacy “Grigore T. Popa”, 700115 Iasi, Romania; 5 Department of Biomedical Science, Faculty of Medical Bioengineering, University of Medicine and Pharmacy “Grigore T. Popa”, 700145 Iasi, Romania; 6 Department of III Internal Medicine Clinic, “St. Spiridon” Emergency County Hospital, 700111 Iasi, Romania; 7 Department of Cardiology, Arcadia Hospital, 700620 Iasi, Romania; 8 Department of Mother and Child Medicine-Pediatrics, University of Medicine and Pharmacy “Grigore T. Popa”, 700115 Iasi, Romania; 9 Department of Pedriatics, Arcadia Hospital, 700620 Iasi, Romania; 10 Department of Internal Medicine III, Faculty of Medicine, University of Medicine and Pharmacy “Grigore T. Popa”, 700145 Iasi, Romania; 11 Department of Cardiology, Helicomed Hospital, 700115 Iasi, Romania

**Keywords:** myocardial infarction, 24 h Holter ECG, T-wave

## Abstract

Myocardial ischemia is a pathophysiological state characterized by inadequate perfusion of the myocardium, resulting in an imbalance between myocardial oxygen demand and supply. It is most commonly caused by coronary artery disease, in which atherosclerotic plaques lead to luminal narrowing and reduced blood flow to the heart. Myocardial ischemia can manifest as angina pectoris or silent myocardial ischemia and can progress to myocardial infarction or heart failure if left untreated. Diagnosis of myocardial ischemia typically involves a combination of clinical evaluation, electrocardiography and imaging studies. Electrocardiographic parameters, as assessed by 24 h Holter ECG monitoring, can predict the occurrence of major adverse cardiovascular events in patients with myocardial ischemia, independent of other risk factors. The T-waves in patients with myocardial ischemia have prognostic value for predicting major adverse cardiovascular events, and their electrophysiological heterogeneity can be visualized using various techniques. Combining the electrocardiographic findings with the assessment of myocardial substrate may offer a better picture of the factors that can contribute to cardiovascular death.

## 1. Introduction

Myocardial ischemia is a pathological condition described by a reduction in blood flow and oxygen supply to the myocardium, resulting in cellular damage and dysfunction [1,2]. Myocardial infarction (MI) is a form of acute coronary syndrome that features a sustained decrease in blood supply to an area of the myocardium, leading to ischemia, necrosis and cellular death [3,4,5].

MI is a prevalent condition that affects millions of people worldwide, responsible for 16% of all deaths globally [2,4]. The risk of myocardial infarction increases with age, and men are more likely to experience the condition than women. Various factors, such as smoking, obesity, hyperlipidemia, or hypertension, can increase the risk of myocardial infarction [5,6]. Early identification and treatment of myocardial infarction are crucial for improving outcomes and reducing mortality, as thrombotic occlusion of a coronary artery typically underlies the condition [5,7]. The degree of myocardial damage, treatment effectiveness, and the presence of comorbidities determine prognosis after myocardial infarction [6,7]. While the incidence of new arrhythmias in myocardial infarction is unknown, left ventricular ejection fraction has been the primary criterion for recommending implantable cardioverter defibrillators to prevent sudden cardiac death [8]. However, there is a need for additional methods to stratify the risk of sudden cardiac death [8,9]. Variability in repolarization characteristics, specifically T-wave morphologies, has been linked to a higher risk of major adverse cardiovascular events during myocardial ischemia. Therefore, extensive research has been conducted in this area [10,11,12].

Thus, we proposed conducting a review of various publications that describe the observed T-wave patterns captured during 24 h Holter ECG monitoring in patients with myocardial ischemia and the risk of developing a major adverse cardiovascular event (MACE), as well as what future perspectives are.

We conducted a thorough search on PubMed and Google Scholar for articles published between 1993 and August 2022 and created a database by using specific keywords: “myocardial ischemia”, “Holter ECG”, “T-wave”, “repolarization”, “T-wave alternans”, “T-wave patterns”, “myocardial infarction”, “acute coronary syndrome”, “major adverse cardiovascular events”, “stroke”, “cardiac magnetic resonance imaging”, “electroanatomic mapping”, “artificial intelligence” and all combinations of them. The findings comprised 257 papers, including various types of research, such as original papers, reports, prospective studies, systematic reviews, retrospective studies, meta-analyses and case reports, which examined T-wave patterns and their relevance to risk stratification in myocardial infarction. We conducted a comprehensive analysis of relevant articles to obtain data and present a detailed overview of the various aspects of the T-wave aspects observed on 24 h Holter ECG monitoring in patients with myocardial infarction who had been followed-up for several years. In our study, we employed exclusion criteria to ensure the integrity of our analysis. Specifically, we omitted papers that lacked patient follow-up, were incomplete or inconclusive, or were pilot studies. Additionally, we excluded reviews that substantially overlapped with our existing findings.

### Overview of Myocardial Infarction and Major Adverse Cardiovascular Events

MI is a clinical condition that results from a prolonged blockage of blood supply to the heart, causing necrosis of the myocardium [1,2,3,4]. Atherosclerotic plaque rupture is the most common cause of MI [2,3,5]. MI contributes significantly to global mortality and morbidity, with approximately 7.8 million fatalities reported annually. The clinical presentation of MI varies but typically includes chest pain, difficulty breathing, diaphoresis, dizziness and vomiting [2,13]. The diagnosis of MI is established through clinical symptoms, electrocardiographic changes and biomarker elevations [4,5,13]. Early diagnosis and treatment are crucial for reducing myocardial damage and improving outcomes [13]. Prognosis after MI depends on the degree of myocardial damage, the presence of comorbidities and different noninvasive ECG aspects that reflect aberrations in depolarization or repolarization, as well as an imbalance in automatic function, which may serve as predictors of an unfavorable outcome [7,14,15,16]. The Holter ECG monitoring recording is a vital diagnostic tool for identifying MI patients who may be susceptible to sudden cardiac death [17].

In cardiovascular research, MACE are commonly used as an endpoint, which consists of both mortality and morbidity among patients who have suffered a myocardial infarction [18,19]. MACE can be defined as a composite endpoint encompassing non-fatal stroke, cardiovascular mortality, non-fatal myocardial infarction, hospitalization due to AHF, ventricular arrhythmia, sudden cardiac death (SCD), or coronary revascularization [18,20,21,22,23,24,25,26,27,28]. SCD is a major public health concern and is defined as a natural death caused by cardiac disease within 24 h of symptom onset or within one hour of symptom onset at the latest in an individual without any underlying medical conditions [29,30,31]. The Holter ECG system can enhance healthcare outcomes due to its greatly improved diagnosis and follow-up for various cardiovascular diseases [32]. Electrocardiographic parameters based on 24 h Holter ECG monitoring have been documented to be independent risk predictors of the progression of myocardial infarction and total mortality [32,33,34,35,36,37,38]. Combining electrocardiographic stratification with an assessment of myocardial substrate may provide a closer look at the factors that can contribute to death [2,39,40,41,42].

## 2. The T-Wave Morphology Documented on the 24 h Holter ECG Recording

The T-wave is an important aspect of the ECG that represents ventricular repolarization, with a standard orientation pattern characterized by inversion in lead aVR and upward deflection in other leads [29,43,44]. T-wave morphology on the 12-lead ECG is prognostic for MACE in myocardial infarction, but excessive electrophysiological heterogeneity in the heart can contribute to arrhythmogenesis and SCD [45,46,47]. The use of Holter ECG monitoring can reveal this heterogeneity through various methods of T-wave analysis [45,47,48]. As such, it is crucial to evaluate ventricular repolarization anomalies to identify myocardial infarction patients at higher risk of cardiac mortality due to SCD [46].

### 2.1. T-Wave Alternans

**Figure 1:**
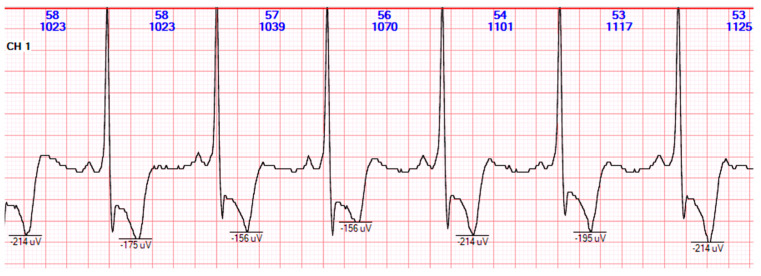
T-wave alternans on 24 h Holter ECG monitoring (Personal collection—recorded with Holter ECG DMS 300-4L).

Cardiac electrical alternans is characterized by alternating changes in the ECG waveform morphology every second cardiac cycle and is typically represented by T-wave alternans (TWA). TWA has been linked to increased susceptibility to SCD in a variety of cardiac pathologies and is considered a valuable risk marker for stratifying the risk of malignant arrhythmia and SCD in patients with structural heart disease [49,50]. Its non-invasive nature makes it a promising tool for this purpose [29,41,51]. TWA has been correlated with dispersion of repolarization and is characterized by beat-to-beat fluctuations in the amplitude, morphology, or duration of the T-wave [29,51,52,53] (Figure 1). The prognostic performance of TWA findings is variable, ranging from highly effective to nearly null, depending on the clinical population and protocol employed [54,55]. TWA analysis of 24 h Holter ECG records provides unique information for arrhythmia risk stratification [56,57]. The pathophysiological mechanism underlying TWA remains incompletely understood, but recent studies have demonstrated a correlation between repolarization alternans and concurrent alterations in intracellular calcium levels [49,58,59,60,61,62,63]. TWA reflects temporal and spatial heterogeneity of repolarization and serves as a measure of electrical instability, which can be utilized for quantification [64,65].

Several studies showed different results for the prediction of MACE in TWA-positive patients [60,66,67].

De Ferrari et al. conducted a meta-analysis in which they incorporated a sample size of nearly 1500 patients and demonstrated that individuals with anomalous TWA exhibit a risk that is three times greater in comparison to those with regular TWA. However, the authors focused on patients with chronic heart failure [60].

The etiology of an acute myocardial pathology is an important aspect because it can influence the results of the TWA recording. The extend of TWA may vary as a result of myocardial disfunction following an acute myocardial infarction [43,66,68]. Arisha et al. demonstrated that over the course of a six-month follow-up period, patients who exhibited positive TWA had an incidence rate of MACE that amounted to 5% [53]. Moreover, Yuan et al. conducted a follow-up study that lasted a minimum of six months in order to document the incidence of MACE among the participants. The statistical analyses conducted in this study did not establish a correlation between alterations in ventricular repolarization, specifically TWA, and the incidence of MACE [25]. However, Ashraf and colleagues performed a statistical analysis that revealed that there was no significant difference in the mean value of TWA and the proportion of patients with positive TWA between those with ischemic and non-ischemic cardiomyopathy. Thus, it can be inferred that the emergence of T-wave alternans in patients with cardiomyopathy and its propensity to give rise to ventricular arrhythmias is not linked to ischemia, since it is comparably evident among individuals with non-ischemic cardiomyopathy [60]. In another investigation, in was observed that over the course of a follow-up period that lasted 1.1 ± 0.6 years, 27 out of the total 295 participants passed away due to cardiac-related reasons. The analysis of TWA resulted in significant hazard ratios in both the subgroups of patients with ischemic and non-ischemic conditions [64].

Another important aspect is the time when the TWA is measured since the acute event, especially in patients with MI. Garcia conducted TWA testing on patients who had experienced an acute MI at least 14 days prior. The results showed that 17% of the tests were positive, 75% were negative and 9% were indeterminate. The study also calculated the sensitivity and negative predictive value of the TWA test for predicting arrhythmic events. Furthermore, when assessing various parameters such as baroreflex sensitivity, TWA and heart rate turbulence within the 2–4 week acute phase following MI, no singular parameter was found to effectively predict outcomes. However, during the non-acute phase (10–14 weeks) after MI, the combination of abnormal TWA and LVEF below 50% yielded the most accurate diagnostic results [54]. A different research investigation demonstrated that TWA has the potential to forecast the prognosis following MI when assessed at roughly 30 days after the incident. However, risk assessment post-MI proves to be a challenging task, particularly in populations with preserved systolic function and concomitantly low incidence of adverse events. The Alternans Cardiac Electrical Stability Study findings indicate that TWA progresses from initial to subsequent evaluations, exhibiting 67% concurrence [52]. The administration of TWA testing prior to patient discharge subsequent to acute MI does not reveal an augmented probability of mortality, showed Tapanainem et al. Despite a mortality rate of 6.9% among the patient cohort during an average follow-up duration of six months and the detection of persistent TWA in 14.7% of patients, no instances of mortality were observed in patients exhibiting positive TWA [67]. Additional research investigations have demonstrated that the occurrence of TWA in the early aftermath of MI is about 25% [63].

Nishibe et al. proposed in their study one potentially valuable metric for assessing alternans, known as Alternans Ratio. The study demonstrated that a set of alternans indices was established for every heartbeat, which comprised the orthogonal waveform distance between the target beat and the neighboring two beats. In the presence of alternans, the first index was found to be greater than the second index [69].

Therefore, TWA meets the fundamental criteria for serving as a therapeutic marker, particularly in individuals diagnosed with myocardial infarction, being the causal pathway of arrhythmogenesis [62]. Historically, TWA has been applied to guide the decision-making process for implanting cardioverter-defibrillators (ICD), whereas its capacity to aid in directing pharmacologic therapy has not been fully recognized [51]. Elevated TWA furnishes supplementary insights for forecasting appropriate implantable cardioverter-defibrillator therapy in myocardial infarction patients because malignant tachycardia episodes have been considered as a surrogate for SCD. Monasterio et al. showed that TWA has been useful in identifying a subgroup of patients with myocardial infarction who are less likely to benefit from ICD therapy. However, there are conflicting results regarding the association between TWA classification and the risk of malignant tachyarrhythmias in patients who have received ICD therapy [70].

Although the existence of macroscopic TWA has been documented, it was regarded as an uncommon discovery, consistently linked to an unfavorable prognosis, until microscopic TWA was initially reported in the 1980s [54].

### 2.2. Microvolt T-Wave Alternans

#### 2.2.1. Definition of Microvolt T-Wave Alternans

**Figure 2:**
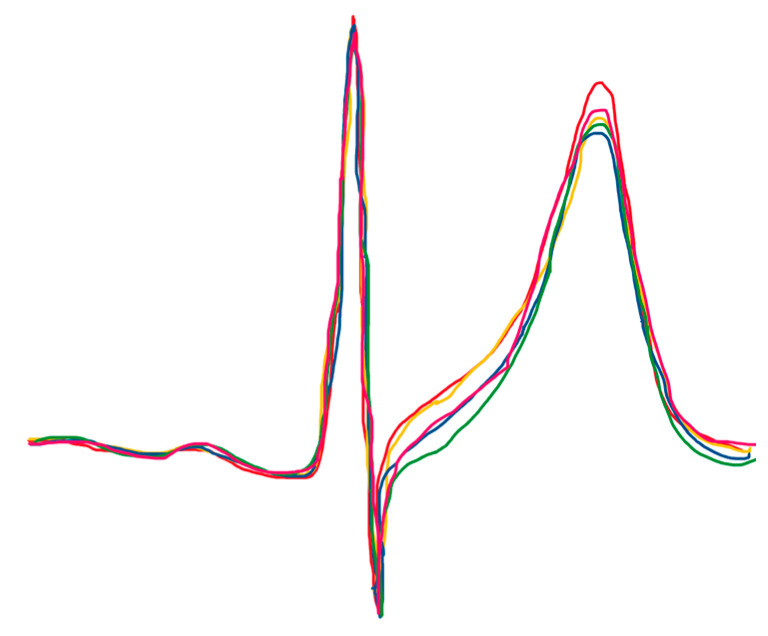
Microvolt T-wave alternans (Personal chart).

Microvolt T-wave alternans (MTWA) is a phenomenon characterized by fluctuations in the form and magnitude of the T-wave, resulting in an oscillatory pattern, and has been shown to be useful in predicting malignant ventricular arrhythmias and SCD in various cardiac disorders [29,41,71,72,73,74,75,76,77,78] (Figure 2).

MTWA reflects beat-to-beat variation in the spatial and temporal repolarization heterogeneity, caused by cellular repolarization alternans, and may provide a substrate for reentry [79,80,81]. The accurate evaluation of MTWA is heavily reliant on meticulous skin preparation to minimize the impedance between the skin and the electrodes. The detection and measurement of MTWA require the application of sophisticated signal processing techniques, primarily due to its small amplitude and susceptibility to background noises [82]. Therefore, new algorithms have been developed for its detection. MTWA may possess distinctive value in detecting a low-risk cohort with a high negative predictive value, especially in individuals with congestive heart failure [29].

#### 2.2.2. The Analysis of Microvolt T-Wave Alternans

The identification of microvolt T-wave alternans (MTWA) can be accomplished through two methodologies: the spectral approach and the modified moving average (MMA) method applied in the time domain [29,72]. Both techniques require specialized equipment and software. The spectral method requires a target heart rate of 105–110 beats per minute for a specific period using specialized exercise protocols, pharmacological agents, or atrial pacing. It also requires the use of exclusive high-resolution electrodes [29,31]. The MMA methodology, conversely, does not require specialized electrodes. The spectral method calculates the alternans voltage by extracting the square root of the spectral power at the alternans frequency, with a minimum MTWA level of ≥1.9 μV required for a positive test result. However, up to 40% of tests are classified as “indeterminate” due to the spectral method’s requirement of achieving a steady target heart rate, which may be impeded by patient-related factors [29,30,31].

In the domain of clinical research for the assessment of Holter ECG MTWA, the preeminent method employed is the non-spectral approach referred to as MMA [41,73,83]. The MMA method is a non-spectral approach that involves segregating odd and even beats into separate compartments and calculating the maximal MTWA value for every 15 s of the ECG recording by determining the maximum discrepancy between successive T-waves [84,85,86,87,88]. This technique enables the evaluation of MTWA in the context of both standard exercise stress testing and 24 h Holter ECG monitoring, obviating the need for specialized electrodes or a regulated heart rate [30,87,89]. The MMA technique has conventional thresholds of 47 μV for an “abnormal” outcome and 60 μV for a “severely abnormal” test result [41,90]. However, current recommendations suggest using the maximum recorded MTWA level during pharmacotherapy, without considering the time of day, heart rate, or ST-segment deviation [91].

MTWA has a high negative predictive value of 98%, but a low positive predictive value of 8–10% in low-risk populations. Studies have shown that the absence of notable MTWA in individuals with myocardial infarction significantly reduces the likelihood of SCD. However, the low positive predictive value of MTWA has led to an interest in exploring a composite of noninvasive parameters, primarily autonomic indices such as heart rate turbulence [74,92,93]. Combining heart rate turbulence with MTWA quantification using either spectral or MMA approaches may improve the classification of the risk of cardiovascular fatality, which aligns with the underlying mechanism of SCD arising from transient triggers acting on an unstable substrate. Further research is needed to determine whether advancements in MTWA detection methodology can enhance its positive diagnostic capacity [93].

#### 2.2.3. The Importance of Microvolt T-Wave Alternans

##### Microvolt T-Wave Alternans Analysed through the Spectral Method

The use of 24 h ambulatory ECG monitoring to evaluate TWA has shown potential in predicting cardiac mortality [91,94,95,96]. Spectral analysis has been widely employed in processing MTWA in patients with chronic heart failure, which enables stratification into low and high-risk groups for SCD [97,98,99,100]. Positive TWA has been associated with non-sustained ventricular tachycardia, ventricular premature complexes and a high risk of malignant tachyarrhythmic events [80,97]. Hennersdorf and colleagues demonstrated a significant association between patients with a history of chronic heart failure and a positive TWA and the occurrence of non-sustained ventricular tachycardia during the six-month follow-up period. In addition, patients with a positive MTWA test exhibited a relatively higher frequency of ventricular premature complexes compared to those who tested negative, although this difference was not statistically significant [97]. Nevertheless, Ikeda and colleagues reported that using the same analytical technique, 49% of patients demonstrated MTWA, with 15% of these individuals experiencing symptomatic, sustained ventricular tachycardia or ventricular fibrillation during a six-month follow-up [80]. The concurrent evaluation of MTWA and late potentials has a strong positive predictive value for an arrhythmic event in patients with myocardial dysfunction following acute myocardial infarction. Noninvasive determination of MTWA, along with abnormal heart rate turbulence and ventricular premature contraction, shows promise in identifying high-risk patients with myocardial dysfunction following acute myocardial infarction [78,80,88].

##### Microvolt T-Wave Alternans Analysed through the Time-Domain Modified Moving Average Method

Given that certain patients, including those receiving medications such as beta-blockers and digoxin, and those with physical constraints, are not suitable candidates for spectral MTWA testing, the majority of studies have concentrated on employing the MMA technique [87]. The presence of MTWA detected using the MMA technique was significantly associated with a nearly three-fold greater risk of cardiac death or resuscitated cardiac arrest during the follow-up period [91,101]. The measurement of MTWA from 24 h Holter ECGs is a predictive factor for cardiac mortality in individuals with ischemic and nonischemic left ventricular dysfunction. In Sakaki’s investigation, 9.2% of participants met the primary endpoint, which was defined as cardiac mortality [102]. Among these patients, five experienced SCD, nine died from congestive heart failure, and twelve died as a result of cardiac complications, such as congestive heart failure and frequent ventricular arrhythmias [87].

Maeda et al. found that in patients with myocardial infarction, a positive MTWA was a significant covariate for the incidence of life-threatening ventricular arrhythmias during a mean follow-up of 6 years [103]. Furthermore, Hoshida et al. demonstrated that MTWA was more closely linked to arrhythmic events than to overall cardiac mortality [104]. Another important aspect described by Ikeda et al. was the right moment for MTWA measurements after an acute episode of myocardial dysfunction, caused by myocardial infarction (see Table 1). The authors proposed that to enhance the prognostic power of MTWA testing, it should be conducted several weeks after MI, preferably at least 2 to 3 weeks later. This is because the measurement of MTWA immediately after acute MI (i.e., 8 days) did not reveal an increased risk for mortality. Subsequent studies have confirmed that MTWA is the most significant predictor for SCD in this patient population [105]. In disagreement with the previous study, Yu et al. studied the period immediately following MI, with MTWA being monitored within 15 days after MI. In their cohort study, MTWA could identify patients with a heightened susceptibility to fetal ventricular arrhythmias [86]. Thus, the predictive utility of MTWA after MI can predict SCD, depending on the time of the investigation and the associated pathologies [31].

Selvaraj et al. observed that while a positive MTWA test indicated a higher risk of cardiac death and life-threatening arrhythmia in both ischemic and nonischemic cardiomyopathy, in the context of patients with myocardial infarction, the incidence of sudden cardiac death was significant, yet no association was found between SCD and MTWA [106,107,108]. In another study that analyzed myocardial dysfunction, MTWA was positive in 17% of patients, during a follow-up of 3 years, but only 1,8% reached an endpoint defined as SCD or life-threatening arrhythmic events [109].

The Eplerone Post-Acute Myocardial Infarction Hear Failure Efficacy and Survival Study (EPHESUS) showed that the amplitude of MTWA was observed to be greater in patients who suffered from sudden cardiac death compared to survivors or those without sudden cardiac death, irrespective of the lead used for measurement. According to research, Holter ECG-based MTWA measured by MMA is a strong predictor of SCD in high-risk post-MI patients with left ventricular dysfunction. The maximum MTWA was higher in patients who suffered sudden cardiac death, but there was no significant difference between survivors and non-SCD patients. The study also noted that the highest levels of MTWA were typically found in the early afternoon, which deviates from the usual circadian pattern of SCD that peaks in the early morning hours. However, this timing aligns with the increased risk of SCD in heart failure patients [110]. Sulimov et al. included in the endpoint, not only SCD or malignant ventricular arrhythmias but also stroke, being one of the few studies that correlates MTWA with stroke. Over a 12-month follow-up period, there were 15 cases of sudden cardiac death and 8 cases of non-sudden cardiovascular death, which included 5 fatal myocardial infarctions and 3 fatal strokes. The individuals who did not survive had significantly higher values of MTWA [111].

The initial trial that utilized MTWA as a tool to direct prophylactic ICD placement is the Alternans Before Cardioverter Defibrillator (ABCD) study. Over a median follow-up period of 1.9 years, a primary endpoint of either appropriate ICD discharge or SCD at one year was met by 7.5% of the patients [112,113]. In a different investigation, MTWA may lack the ability to detect a subset with a low enough risk in this cohort to eliminate the requirement for prophylactic ICD placement, despite total mortality being lower in the MTWA-negative cohort [114]. Conversely, MTWA has the potential to differentiate not only a high-risk cohort but also a low-risk group who are unlikely to gain advantages from ICD prophylaxis and could survive for two or more years without enduring death or persistent ventricular arrhythmia [115].

Assessment of myocardial substrate pertains to the evaluation of the underlying structural and functional abnormalities of the heart, which can precipitate arrhythmias and sudden cardiac death. This includes conditions such as ischemic heart disease, cardiomyopathy, heart failure, and valvular heart disease [116,117]. Diagnostic tools such as echocardiography, cardiac magnetic resonance imaging, or invasive electrophysiological testing can be used to assess myocardial substrate. TWA has been identified as a valuable marker for identifying patients at heightened risk of arrhythmic events, particularly in those with structural heart disease such as ischemic cardiomyopathy or dilated cardiomyopathy [118,119,120]. In these patients, TWA has been shown to be a more potent predictor of arrhythmic events than conventional risk factors such as left ventricular ejection fraction or QRS duration [119,121].

The combination of TWA and assessment of myocardial substrate has been demonstrated to improve the risk stratification for arrhythmic events and sudden cardiac death compared to using either parameter alone [120,121]. For instance, a study published in the Journal of the American College of Cardiology in 2009 showed that combining TWA with left ventricular scar burden, a measure of myocardial substrate, was a superior predictor of arrhythmic events compared to either parameter alone [122,123,124]. Another study discovered that TWA, a marker of repolarization instability on the ECG, was independently associated with both left ventricular systolic and diastolic dysfunction on echocardiography and predictive of future MACE in patients with non-ischemic cardiomyopathy [125,126]. There is evidence to indicate that T-wave aspects and ejection fraction (EF) may have complementary predictive value for MACE in patients with cardiovascular disease. In a study of patients with left ventricular systolic dysfunction, TWA was a stronger predictor of all-cause mortality compared to EF. Patients with TWA had a considerably higher risk of mortality regardless of EF [127].

In a study of patients with ischemic cardiomyopathy and abnormal electrograms on electroanatomic mapping, both TWA and left ventricular EF were found to be independent predictors of MACE. Patients with both TWA and EF ≤ 30% had the highest risk of MACE, followed by those with TWA or EF ≤ 30% alone [128]. Additionally, in a study of patients with non-ST-elevation myocardial infarction, T-wave inversion on the initial ECG was a significant predictor of adverse cardiac events at 1 year, even after adjusting for EF. Patients with T-wave inversion and EF < 40% had the highest risk of adverse events [129]. Furthermore, in a study of patients with chronic stable angina and left ventricular dysfunction, T-wave amplitude and QT interval were independent predictors of cardiac events and were particularly useful for risk stratification in patients with EF ≤ 35% [130]. The combination of T-wave amplitude and QT interval had a higher predictive value for MACE compared to either parameter alone. Another study of patients with coronary artery disease and left ventricular dysfunction found that TWA was a strong predictor of all-cause mortality, particularly in patients with EF ≤ 35% [131]. Adding TWA to clinical and echocardiographic variables significantly improved risk stratification for mortality. Lastly, in a study of patients with acute ST-segment elevation myocardial infarction, T-wave inversion on the initial ECG was a strong predictor of one-year mortality, particularly in patients with EF ≤ 40%. The combination of T-wave inversion and EF ≤ 40% had the highest risk of mortality [132]. Collectively, these results suggest that T-wave patterns on the ECG and echocardiographic parameters are closely associated and can offer complementary information for risk stratification in patients with cardiovascular disease [128,129,130,131,132].

Several studies have explored the predictive value of both MTWA and myocardial scars for MACE. For instance, a 2017 study in the Journal of the American Heart Association investigated the prognostic value of TWA and late gadolinium enhancement (LGE) on cardiac magnetic resonance imaging (MRI) for predicting MACE in patients with nonischemic cardiomyopathy [133,134,135]. The study found that the presence of both TWA and LGE was associated with an increased risk of MACE and that the combination of TWA and LGE had a higher predictive accuracy for MACE than either TWA or LGE alone (C-index 0.78 vs 0.66 and 0.72, respectively) [136].

Similarly, a 2016 study in the Journal of the American College of Cardiology assessed the prognostic value of T-wave inversion (TWI) and LGE on cardiac MRI for predicting MACE in patients with hypertrophic cardiomyopathy (HCM). The study found that the presence of both TWI and LGE was associated with a higher risk of MACE compared to either TWI or LGE alone (hazard ratio 6.7 vs 2.5 and 3.6, respectively) [121].

Several studies have investigated the prognostic value of combining T-wave aspects and myocardial scars for predicting MACE in different patient populations [137,138,139]. For instance, in a 2015 study published in JACC: Cardiovascular Imaging, researchers assessed the predictive value of TWA and LGE on cardiac MRI for MACE in patients with dilated cardiomyopathy. They found that the presence of both TWA and LGE was associated with an increased risk of MACE and that the combination of TWA and LGE had a higher predictive accuracy for MACE compared to either TWA or LGE alone (C-index 0.80 vs 0.69 and 0.70, respectively) [140,141,142].

In addition, in patients with ischemic heart disease, T-wave patterns on the ECG and findings on CMR have been found to correlate with MACE. TWA has also been identified as a predictor of future MACE in patients with ischemic heart disease [143].

While these findings suggest that combining T-wave patterns and myocardial scars on cardiac MRI may provide valuable information for risk stratification in certain patient populations, more research is needed to validate these findings and determine their clinical relevance [143,144].

TWA has been associated with myocardial ischemia and infarction. In a study involving patients with chronic stable angina, TWA was found to be a predictor of myocardial ischemia on CMR and was also linked to a higher risk of major cardiac events during follow-up [144].

In addition to T-wave patterns, other ECG parameters such as QRS duration and QT interval have also been found to correlate with CMR findings in ischemic heart disease. For example, prolonged QRS duration has been associated with a higher incidence of myocardial fibrosis on CMR, as well as an increased risk of MACE [145]. Similarly, prolonged QT interval has been linked to a higher prevalence of myocardial edema on CMR, and has also been correlated with an elevated risk of MACE [146].

Taken together, these findings underscore the value of integrating ECG parameters with CMR imaging to stratify the risk of patients with ischemic heart disease [143,144,145,146].

Electroanatomic mapping (EAM) is a non-invasive imaging technique that generates a 3D map of the heart’s electrical activity and is widely utilized in the management of arrhythmias. Although limited research has been conducted on the association between T-wave patterns and EAM findings in predicting MACE, some studies have provided insights into this relationship. For instance, in patients with ventricular tachycardia and structural heart disease, the presence of TWA on the surface ECG was linked to an elevated probability of identifying critical isthmus sites on EAM, defined as the location of the slow conduction that sustained the VT. Furthermore, patients with TWA were found to have a higher risk of recurrent VT during follow-up [147]. Similarly, in patients with ischemic cardiomyopathy, the presence of TWA on the ECG was associated with a higher probability of detecting abnormal electrograms on EAM within the infarcted myocardium. These electrograms were characterized by low-amplitude, high-frequency signals indicative of scar tissue or conduction block. Notably, patients with both TWA and abnormal electrograms were at a higher risk of MACE during follow-up [128].

A study conducted on patients with nonischemic cardiomyopathy revealed that the presence of TWA on the ECG was linked with abnormal local activation time (LAT) on EAM in the right ventricular outflow tract. This abnormal LAT was characterized as a delay in the timing of the electrical signal relative to neighboring sites, indicating slow conduction or conduction block. Patients with TWA and abnormal LAT had a higher probability of developing sustained ventricular arrhythmias [148]. These studies suggest that the occurrence of T-wave patterns on the ECG might be linked with abnormal electrical activity in the heart as detected by EAM, which may increase the risk of major adverse cardiovascular events. However, further research is needed to confirm these findings and assess the clinical usefulness of integrating ECG and EAM data for risk stratification in patients with cardiovascular disease [128,147,148].

According to several studies, TWA on the Holter ECG/24 h monitoring is associated with abnormal electrograms on EAM in the region of the infarcted myocardium in patients with ischemic cardiomyopathy or heart disease. Abnormal electrograms are defined as low-amplitude, high-frequency signals indicative of scar tissue or conduction block, or as fragmented electrograms with complex waveforms suggesting conduction abnormalities [128]. Delayed electrical activation in the region of the infarcted myocardium has also been associated with the presence of TWA on the ECG in patients with acute myocardial infarction [149]. Patients with TWA and abnormal electrograms, delayed activation, or fragmented electrograms have a higher risk of developing ventricular arrhythmias and MACE [150]. These findings suggest that combining ECG and EAM data may be useful for risk stratification in patients with ischemic heart disease. However, more research is needed to confirm these findings and evaluate the clinical utility of this approach [128,149,150].

There is a scarcity of research that directly compares the predictive value of T-wave features with residual ischemia detected through perfusion imaging in forecasting MACE. In one study that evaluated patients with stable coronary artery disease, TWA was identified as an independent predictor of cardiac events, including MACE, over a follow-up period of 2.2 years. The inclusion of TWA in clinical and myocardial perfusion imaging variables enhanced the prediction of MACE, but the study did not explore the role of residual ischemia on perfusion imaging specifically [151]. In contrast, another study that assessed patients with acute myocardial infarction discovered that T-wave amplitude and TWA on the initial ECG were strong predictors of 30-day MACE. However, adding residual ischemia on perfusion imaging to clinical and ECG variables did not result in a significant improvement in risk prediction [152]. Similarly, in a study of patients with stable angina and suspected coronary artery disease, T-wave amplitude and TWA were determined as independent predictors of MACE, but the inclusion of myocardial perfusion imaging variables did not significantly enhance the prediction of risk [127]. Overall, these studies imply that T-wave features, particularly T-wave amplitude and TWA, may possess prognostic value for MACE in patients with ischemic heart disease. Nonetheless, the role of residual ischemia on perfusion imaging in risk prediction remains less clear [127,151,152].

Several studies have investigated the prognostic value of T-wave aspects in conjunction with coronary angiography for the prediction of MACE. In a study by Ghanbari et al., 603 patients with stable coronary artery disease who underwent coronary angiography were monitored for a median of 2.6 years. TWA was measured using a modified moving average method during exercise treadmill testing, while the severity of coronary artery disease was assessed via angiography. The results showed that TWA was a significant independent predictor of MACE, although the addition of angiographic variables did not significantly improve risk prediction beyond TWA alone [153].

In a study by Haigney et al. (2004), 219 patients with stable coronary artery disease who underwent coronary angiography were followed for a mean of 16 months. T-wave amplitude and TWA were measured during exercise treadmill testing, while the extent and severity of coronary artery stenosis were assessed using the Gensini score. The study found that T-wave amplitude and TWA were significant predictors of cardiac events, independent of the Gensini score and other clinical variables. However, the contribution of residual ischemia on angiography to risk prediction was not specifically examined [151].

The studies conducted by Schuster et al., Verrier et al. and Maytin et al. investigated the predictive value of T-wave aspects, particularly TWA, in patients with stable angina or coronary artery disease [154,155]. In Schuster et al.’s study, 139 patients underwent both TWA testing and coronary angiography to assess the complexity and severity of coronary artery disease using the Syntax score. During the 22-month follow-up, 11 cardiac events occurred, and TWA was found to be a significant predictor of cardiac events, independent of the Syntax score and other clinical variables. However, adding the Syntax score did not significantly improve risk prediction beyond TWA alone. In Verrier et al.’s study, 186 patients with stable coronary artery disease were followed for 25 months after undergoing TWA testing during exercise treadmill testing and coronary angiography to assess the presence and severity of coronary artery disease. During follow-up, 23 MACE events occurred, and TWA was found to be a significant predictor of MACE, independent of angiographic and clinical variables [154]. Similarly, in Maytin et al.’s study, 605 patients with stable coronary artery disease were followed for a median of 3.5 years after undergoing TWA testing during exercise treadmill testing and coronary angiography to assess the severity of coronary artery disease using the Gensini score. During follow-up, 103 MACE events occurred, and TWA was found to be a significant predictor of MACE, independent of the Gensini score and other clinical variables [155]. These studies suggest that T-wave aspects, particularly TWA, may be useful in predicting MACE in patients with stable or unstable coronary artery disease, independent of the severity of coronary artery disease assessed by coronary angiography or other clinical variables. However, more research is needed to determine the optimal use of T-wave aspects in risk stratification and clinical decision-making [154,155].

The studies conducted by Schuster et al., Verrier et al. and Maytin et al. investigated the predictive value of T-wave aspects, particularly TWA, in patients with stable angina or coronary artery disease [154,155]. In Schuster et al.’s study, 139 patients underwent both TWA testing and coronary angiography to assess the complexity and severity of coronary artery disease using the Syntax score. During the 22-month follow-up, 11 cardiac events occurred, and TWA was found to be a significant predictor of cardiac events, independent of the Syntax score and other clinical variables. However, adding the Syntax score did not significantly improve risk prediction beyond TWA alone. In Verrier et al.’s study, 186 patients with stable coronary artery disease were followed for 25 months after undergoing TWA testing during exercise treadmill testing and coronary angiography to assess the presence and severity of coronary artery disease. During follow-up, 23 MACE events occurred, and TWA was found to be a significant predictor of MACE, independent of angiographic and clinical variables [154]. Similarly, in Maytin et al.’s study, 605 patients with stable coronary artery disease were followed for a median of 3.5 years after undergoing TWA testing during exercise treadmill testing and coronary angiography to assess the severity of coronary artery disease using the Gensini score. During follow-up, 103 MACE events occurred, and TWA was found to be a significant predictor of MACE, independent of the Gensini score and other clinical variables [155]. These studies suggest that T-wave aspects, particularly TWA, may be useful in predicting MACE in patients with stable or unstable coronary artery disease, independent of the severity of coronary artery disease assessed by coronary angiography or other clinical variables. However, more research is needed to determine the optimal use of T-wave aspects in risk stratification and clinical decision-making [154,155].

A study by Baman et al. followed 1149 patients with acute coronary syndrome who underwent coronary angiography for a mean of 3.8 years. TWA was measured using the spectral method during exercise treadmill testing, while coronary angiography was utilized to determine the presence and severity of coronary artery disease. During the follow-up period, there were 311 MACE events, including cardiac death, nonfatal myocardial infarction, and revascularization. TWA was found to be a significant predictor of MACE, even after adjusting for angiographic and clinical variables [155].

In summary, these studies indicate that T-wave parameters, particularly TWA, may be valuable in predicting MACE in patients with stable or unstable coronary artery disease, regardless of coronary angiography results. Nevertheless, further research is required to establish the optimal application of T-wave parameters in clinical decision-making and risk stratification [68,154,155].

The SYNTAX score is a tool utilized to evaluate the complexity of coronary artery disease, based on the severity, location, and number of stenoses present in the coronary arteries. It is frequently employed to inform decision-making for coronary revascularization procedures, such as percutaneous coronary intervention (PCI) or coronary artery bypass grafting (CABG). While the SYNTAX score has demonstrated predictive value for adverse cardiovascular events, it is not typically used in conjunction with T-wave patterns as a predictor [156].

Nevertheless, there is some research suggesting that T-wave patterns may be helpful in predicting outcomes following revascularization procedures. For instance, one study found that T-wave alternans was linked to worse outcomes following PCI in patients with stable angina. Another study found that the presence of abnormal T-wave morphology was associated with higher rates of major adverse cardiac events after CABG [157].

Although the SYNTAX score has demonstrated some predictive value for adverse cardiovascular events, there is limited research on combining T-wave patterns and the SYNTAX score to predict outcomes. Further studies are necessary to determine the potential utility of combining these factors in predicting major adverse cardiovascular events [156,157].

## 3. Future Perspectives

The presence of inhomogeneous repolarization has been linked to an elevated likelihood of ventricular arrhythmias. T-wave morphologies have been extensively researched over the past few decades in association with this phenomenon [10]. Multiple studies have provided evidence that various abnormalities of the T-wave are linked with increased susceptibility to MACE among individuals with acute myocardial infarction. We found in the literature different analyzes regarding the T-wave, known under multiple names: started with the inverted T-wave (TWI) to nonspecific T-wave: T-wave heterogeneity (TWH), biphasic T-wave, T-wave loop morphology [11,12,158,159]. Most of the authors have shown in their studies that those T-wave aspects are correlated with a higher risk of SCD, malignant arrhythmias, myocardial infarction, stroke, overall mortality, or rehospitalization for AHF [3,11,118,119,120,160,161]. Multiple elements of repolarization heterogeneity as a potential etiology of ventricular arrhythmogenesis will be discussed [3,4,162,163,164,165,166,167,168,169,170,171,172,173,174,175,176,177].

Recent research has investigated the application of advanced techniques, such as wavelet transform and principal component analysis, for analyzing T-wave morphology in order to enhance the accuracy of predicting MACE. These techniques enable more precise measurements of various T-wave attributes, including amplitude, duration, and shape, and can detect subtle changes in these features to improve the accuracy of MACE prediction [178,179].

T-wave discordance refers to the incongruity between the T-wave direction and the QRS complex direction observed on an ECG or Holter ECG/24 h monitoring. Such disparity is indicative of ventricular repolarization heterogeneity and is linked to an increased risk of ventricular arrhythmias and sudden cardiac death. Some studies have demonstrated that T-wave discordance can also forecast MACE in individuals with acute coronary syndrome [180,181].

Furthermore, machine learning algorithms have been developed to evaluate T-wave patterns and predict MACE. These algorithms utilize vast datasets to recognize patterns and correlations that may be overlooked by conventional statistical methods. Several studies have reported that machine learning algorithms can effectively predict MACE in patients with heart failure and other cardiovascular diseases based on T-wave characteristics [182,183].

In patients with myocardial disfunction following a myocardial infarction, the overall cosine of the angle formed between the wavefront of depolarization and repolarization is represented by the term Total Cosine R-to-T (TCRT), yielded an independent predictive value for MACE [164]. In an investigation analyzing patients with myocardial dysfunction subsequent to myocardial infarction in comparison to a cohort of individuals with previous ventricular tachycardia, the total cosine R-to-T and the percentage of loop area demonstrating T-wave irregularity were evaluated to determine the global angle formed by the repolarization and depolarization loops. The investigation revealed notable variations in the morphology of repolarization signals in patients with ischemic heart disease who have or have not suffered from ventricular fibrillation and ventricular tachycardia. The study further highlights that the primary cause of T-wave abnormality is associated with the proarrhythmic substrate, rather than being a result of ischemic or ischemic heart disease [165].

However, there is a greater abundance of information available in the literature about the T-wave aspects in patients with chronic heart failure that showed a correlation with MACE and that needed to be studied in patients with an acute myocardial infarction [166,167,168,169,170,171,172,173,174,175,176,177] (see Table 2).

Nearing et al. analyzed the TWH within a time frame of 30 to 45 min prior to the initiation of ventricular tachycardia, as an alternative phrasing. The researchers reached a conclusion that crescendos exist in the level of electrical instability that may serve as early indicators of the onset of non-sustained ventricular tachycardia. They observed that in 91% of patients, the elevated levels of T-wave heterogeneity persisted prior to the initiation of non-sustained ventricular tachycardia, thereby indicating that alterations in T-wave heterogeneity may potentially serve as premonitory signs of heightened cardiac electrical instability. It is noteworthy, however, that the authors have not published any specific investigations focusing on patients with myocardial infarction [12,166,167].

Kumar et al. focused on isolated minor T-wave irregularities, represented by flat or minimally inverted T-waves, measuring less than 1 mm amplitude. The study conducted a 15-year follow-up of the patients, revealing that a positive finding for minor T-wave abnormalities was linked to a significantly elevated risk of primary arrhythmic death, but no significant association was found between such findings and the incidence of nonfatal myocardial infarction [169]. According to another investigation, minor T-wave abnormalities can be regarded as indicators of an elevated risk for cardiovascular mortality in both genders, and have a lasting prognostic impact [170].

The T-wave morphology restitution (TMR) method analyzes the T-wave morphology in association with the heart rate. The quantification of TMR involved the use of time-warping metrics to measure the extent of morphological variation in the T-wave for each RR increment. The results indicated a statistically significant increase in TMR values among SCD victims in comparison to other patients. Therefore, a correlation between T-wave morphology and other aspects, such as heart rate, could better quantify the risk of SCD [171]. However, although TWR was significantly higher in non-survivors, it was not predictive of outcome, defined as SCD, in Smetana’s study [172]. Other T-wave parameters that depend on the heart rate are: T-wave morphologic dispersion, the temporal variability of T-wave morphologic heterogeneity, periodic repolarization dynamics and T-wave area dispersion. However, Ramirez and colleagues introduced a novel index called T-wave Morphologic Variation (TMV), which measures the extent of T-wave morphological variation in relation to a normal reference using a single-lead ECG and a single beat. The authors evaluated the predictive value of TMV and found that it remained significantly associated with MACE. However, the association between TMV and all-cause mortality was no longer significant [171].

T-wave loop morphology represents repolarization abnormalities and is strongly correlated with subsequent onset of myocardial infarction [159]. Moreover, aberrations in the repolarization process, as assessed by T-wave loop analysis, serve as a prognostic indicator for cardiovascular mortality in both genders [173]. Under a different name, but approximately with the same method of measurement as T-wave loop morphology, Huang and colleagues introduced the concept of T-wave morphology dispersion (TMD), a metric that quantifies the heterogeneity of spatial T-wave patterns across different leads by computing the mean angles between all conceivable pairs of reconstruction vectors. By analyzing TMD, the authors were able to refine the assessment of the likelihood of mortality due to cardiovascular events in the participants of the study [46].

In Rahola’s investigation, which involved a mean follow-up duration of 8.6 years, a proportion of 3.9% of the patients suffered sudden cardiac death (SCD) or received resuscitation following a sudden cardiac arrest. The measure of TMD evinced a robust correlation with the risk of SCD and was significantly elevated in patients with a history of SCD relative to their living counterparts. However, TMD did not demonstrate statistically significant differences between those who experienced non-SCD or non-cardiac death and the living population. As such, the temporal fluctuations in electrocardiographic spatial heterogeneity of repolarization, as gauged by TMD, can independently prognosticate the long-term risk of SCD in individuals diagnosed with myocardial infarction [173].

**Figure 3:**
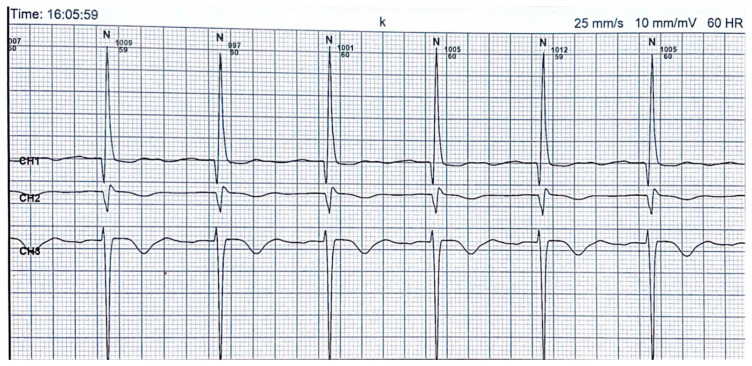
Inverted T-wave on 24 h Holter ECG monitoring (The repository of the Cardiology Clinic of “St. Spiridon” Hospital, Iasi).

The conventional definition of TWI involves the presence of a negative T-wave that exceeds or equals 1 mm in depth in at least two adjacent leads while omitting leads aVR, III and V1 from the evaluation [174]. TWI is a Holter electrocardiography abnormality that may lack specificity or sensitivity. Alternatively, it may serve as a useful biomarker for detecting and diagnosing myocardial infarction or ischemia [160,175,176] (Figure 3).

Krittayaphong’s research identified TWI in 20% of the study population, with 4.4% of patients experiencing MACE. However, the study found no significant interplay between the presence of TWI, whether in conjunction with ST-segment depression or as an independent feature, and the likelihood of MACE [175].

Although T-wave inversion predicted SCD or malignant ventricular arrhythmias, an important observed issue was the inversion of the T-wave in the anterolateral leads, which may be linked to arrhythmogenic cardiomyopathy. Therefore, extended studies that exclude this pathology and include only patients with myocardial infarction are needed [177].

Research indicates that abnormal T-wave patterns can indicate underlying cardiac pathology and increase the risk of cardiovascular mortality. Specifically, T-wave inversion in leads V1-V3 has been identified as a predictor of sudden cardiac death in individuals with hypertrophic cardiomyopathy, whereas T-wave inversion in the inferior leads has been linked to an elevated risk of cardiovascular events in patients experiencing acute coronary syndrome [118,185].

As we mentioned, the combination of T-wave patterns and myocardial substrate assessment has been suggested to improve risk stratification for cardiovascular events and sudden cardiac death compared to either parameter alone. For instance, a 2016 study in the Journal of the American College of Cardiology demonstrated that T-wave inversion in leads V1-V3 and late gadolinium enhancement on cardiac magnetic resonance imaging were a superior predictor of adverse cardiovascular events than each parameter alone in patients with hypertrophic cardiomyopathy [186]. Additionally, another study found that T-wave inversion in leads V1-V4 was associated with reduced LVEF and a higher risk of MACE in patients with idiopathic dilated cardiomyopathy [187]. Moreover, T-wave inversion in leads V1-V3 on the electrocardiogram has been associated with left ventricular diastolic dysfunction and increased left ventricular mass index on echocardiography, which are established risk factors for MACE. However, further research is required to validate these findings and establish the optimal approach for assessing T-wave characteristics and myocardial substrate [188].

In patients with ischemic heart disease, the presence of T-wave inversion on the ECG or Holter ECG/24 h monitoring and the findings on cardiac magnetic resonance imaging (CMR) have been demonstrated to correlate with MACE. Studies have shown that T-wave inversion in leads V1 to V3 on the ECG is associated with the presence of myocardial scarring on CMR in patients with previous MI and is a predictor of future MACE [189]. Furthermore, the extent of T-wave inversion in leads V2 and V3 on the ECG is associated with the extent of myocardial scarring on CMR in patients with previous MI and is an independent predictor of future MACE [190]. In patients with non-ST-segment elevation myocardial infarction (NSTEMI), T-wave inversion in leads V1 to V3 on the ECG is associated with a higher prevalence of myocardial necrosis on CMR, as well as a higher risk of MACE during follow-up [191]. Additionally, T-wave inversion in leads V1 to V4 on the ECG is associated with the presence of microvascular obstruction and intramyocardial hemorrhage on CMR in patients with acute MI [192]. These findings suggest that T-wave patterns and echocardiographic features are closely related and can provide complementary information for risk stratification in patients with cardiovascular disease [189,190,191,192].

Hence, in Pirkola’s study, T-wave area dispersion (TWAD), T-wave morphology dispersion (TMD) and temporal complexity of repolarization (TCRT) showed a substantial correlation with cardiac mortality, with these parameters being more strongly linked with non-sudden cardiac deaths than with SCD. The parameters related to T-wave morphology, which represent the heterogeneity of repolarization, enhance the predictive capacity of the clinical risk model for myocardial infarction in the present treatment era [193].

Artificial intelligence (AI) is a computer-based technology that simulates human intelligence and performs tasks that typically require human cognition, such as visual perception, speech recognition, and decision-making. In the field of cardiovascular disease, AI has shown promise in enabling more accurate and efficient diagnosis and treatment. AI has been applied to various aspects of cardiovascular disease, including risk prediction, image analysis and drug discovery [182,183,194,195]. With respect to predicting major adverse cardiovascular events (MACE) using T-wave aspects as a feature, there is limited research on the use of AI. However, some studies have explored the use of machine learning and AI in predicting MACE in patients with cardiovascular disease. For instance, one study used a deep neural network to predict TWA from standard 12-lead ECGs and achieved high accuracy in predicting TWA compared to traditional spectral analysis methods [182,183]. Another study used machine learning to identify patients with heart failure and reduced ejection fraction who were at high risk of sudden cardiac death by using a combination of clinical variables, including TWA, to predict MACE [183,194]. Additionally, machine learning algorithms have been used to analyze echocardiographic images and clinical data to predict cardiovascular events in patients with suspected coronary artery disease, and deep learning has been used to analyze ECG data and predict cardiovascular outcomes in a large population-based cohort [194,195]. In these studies, the AI models have shown good predictive performance in identifying individuals at high risk of MACE [182,183,194,195].

While there is limited research specifically on the combination of T-wave aspects and AI for predicting MACE, these studies suggest that machine learning and AI may have potential for predicting cardiovascular events in patients with known or suspected cardiovascular disease [194,195]. A deep neural network was employed in a study published in Circulation in 2020 to analyze coronary computed tomography angiography (CCTA) scans and predict MACE in patients with suspected or known coronary artery disease. The authors reported that their model had superior accuracy compared to traditional risk scores in predicting MACE [196]. In a separate study published in JAMA Cardiology in 2018, a machine learning algorithm was used to analyze electronic health record data and predict cardiovascular events in patients with heart failure. The authors reported that their model demonstrated good accuracy in predicting MACE, such as hospitalizations and deaths due to cardiovascular causes [197]. In another study published in Circulation in 2017, a machine learning algorithm was employed to analyze ECG data and predict cardiovascular outcomes in a large population-based cohort. The authors found that their model had good predictive performance in identifying individuals at high risk of MACE, including sudden cardiac death [182,183]. T-wave patterns on an ECG have been suggested as a potentially useful tool for predicting MACE, although further research is needed to validate these findings and determine the optimal methods for analyzing T-wave characteristics. Successful use of T-wave patterns in accurately predicting MACE could have significant implications for the prevention and management of cardiovascular disease [182,183,195].

The prognostic value of T-wave morphology assessed by 24 h Holter ECG monitoring is paramount in identifying patients at risk for MACE. As such, preemptive therapeutic interventions may be warranted for those displaying aberrant T-wave patterns [66]. The deployment of ICD as a prophylactic measure against arrhythmic SCD has become commonplace, yet the prevalence of SCD has not declined despite a steady increase in ICD implantation rates over the past two decades. While non-sustained ventricular tachycardia, ventricular ejection fraction and electrophysiology study-induced programmed stimulation have a high diagnostic yield in identifying individuals at risk for arrhythmic SCD, these markers do not comprehensively account for the population vulnerable to aborted SCD [184].

Patients with LVEF less than 35% are at an increased risk of ventricular arrhythmias, which may require ICD placement. The decision to implant an ICD is based on multiple factors, such as LVEF, history of ventricular arrhythmia, underlying structural heart disease, and certain genetic conditions, to assess the patient’s risk of sudden cardiac death. Several studies have attempted to identify predictors of ventricular arrhythmias in these patients, including T-wave alternans, QT dispersion and heart rate variability [198,199,200,201,202].

Although T-wave patterns alone are not sufficient to warrant ICD implantation, the analysis of T-wave morphology can assist in the decision-making process for ICD implantation in specific clinical scenarios. The current clinical guidelines recommend ICD implantation for primary prevention in patients with reduced EF due to prior MI, nonischemic dilated cardiomyopathy, or genetic or acquired arrhythmogenic disorders associated with an increased risk of sudden cardiac death [199,200,201].

The decision to implant an ICD typically involves multiple factors, such as clinical history, physical examination, imaging studies, electrophysiological testing and risk stratification tools. T-wave patterns and other electrocardiographic findings may be utilized as adjunctive tools to refine risk stratification in certain clinical situations, but they are not considered the sole criterion for ICD implantation [203,204,205].

It is crucial to acknowledge that the decision to implant an ICD is complex and should be tailored to each patient based on their individual clinical characteristics and risk factors. Therefore, patients with suspected or established cardiac disease should undergo a comprehensive evaluation by a qualified cardiologist to determine the most appropriate management plan [199,200,201,202,203,204,205].

The current study results propose that T-wave morphology may be effectively employed to discern a subgroup of patients who are unlikely to derive significant therapeutic benefit from ICD therapy, although further confirmatory investigations are required before endorsing modifications to existing treatment guidelines [66]. Despite this therapeutic approach, because effective antiarrhythmic agents suppress T-wave alternans or other T-wave aspects, the T-wave analysis on 24 h Holter ECG could be a potential application in drug testing [164].

## 4. Conclusions

The repolarization heterogeneity, observed through the different aspects of the T-wave patterns measured on Holter ECG monitoring, is a potent predictor of unfavorable outcomes and significant adverse cardiovascular events in patients with myocardial infarction. Nevertheless, several T-wave aspects showed a correlation with MACE in various cardiovascular pathologies and need to be studied also in patients with myocardial infarction. Because in myocardial infarction the electrical instability can interfere with the results, future studies should focus on the T-wave analysis by finesse methods, which remove most of the artifacts and electrical instability and create a universal analysis algorithm. Not least, AI is a promising tool for research, offering improved accuracy and efficiency in data analysis, identifying complex patterns and supporting informed decision-making processes.

## Figures and Tables

**Table 1:** The moment for microvolt T-wave alternans measurement after myocardial infarction [86,105].

Article	The Moment for MTWA ^1^ Measurement	Year	Number of Participants	Follow-Up Period	Events	The Odds Ratio between MTWA and MACE ^2^
Ikeda et al. [105]	2–3 weeks after the acute heart failure episode	2002	850	2 weeks–1 month	Sudden cardiac death	<0.0001
Yu et al. [86]	Within 1 to 15 days after the acute heart failure episode	2012	227	16 ± 7 months	Lethal ventricular arrhythmias	<0.0001

^1^ MTWA = microvolt T-wave alternans. ^2^ MACE = major adverse cardiovascular events.

**Table 2:** Other T-wave aspects proved to have a correlation with MACE that can also be studied in patients with myocardial infarction [166,167,168,169,170,171,172,173,174,175,176,177].

T-Wave Aspect	Article	Year	Number of Participants	Follow-Up Period	Events	The Odds Ratio between T-Wave Aspect and MACE ^1^
T-wave heterogeneity	Nearing et al. [12]	2012	255	30–45 min before the onset of ventricular tachycardia	Ventricular tachycardia	<0.05
T-wave loop morphology	Okin et al. [172]	2002	1839	3.7 ± 9 years	Cardiovascular death (myocardial infarction, stroke, sudden cardiac death)	<0.0001
The total cosine R-to-T	Rahola et al. [184]	2021	1678	8.6 ± 2.3 years	Sudden cardiac death, Sudden cardiac arrest	<0.03
Minor T-wave abnormalities	Kumar et al. [168]	2008	3224	10 years	Increased risk for arrhythmic death	<0.001
	Greenland et al. [169]	2003	39.573	22 years	Cardiovascular heart disease, especially coronary diseases in men	<0.05
T-wave morphology restitution	Ramirez et al. [170]	2022	23.962	5 years	Sudden cardiac death	<0.001
T-wave morphology dispersion	Huang et al. [46]	2009	650	2.7 ± 1.8 years	Cardiovascular mortality	0.016
	Zabel et al. [173]	2000	280	32 ± 10 months	Ventricular tachycardia, all cause-mortality	<0.001
Inverted T-wave	Kryttayaphong et al. [165]	2019	2009	One year	Cardiovascular mortality, hospitalization resulting from unstable angina or heart failure, non-fatal myocardial infarction	<0.001
	Merlo et al. [175]	2019	414	125 months	Sudden cardiac death, ventricular arrhythmias with malignant potentials, heart transplant	0.041

^1^ MACE = major adverse cardiovascular events.
